# Baicalein links macrophage M2 polarization with reduced synovial inflammation to alleviate gouty arthritis

**DOI:** 10.3389/fimmu.2026.1812532

**Published:** 2026-04-23

**Authors:** Mingli Han, Xianshun He, Longfei Han, Kun Lin, Guifeng Luo, Long Tian, Meixia Li, Shun Lu, Hai Su, Wenyuan Hou, Junjiao Zhang, Mincong He, Fan Yang, Qiushi Wei

**Affiliations:** 1 Guangzhou University of Chinese Medicine, Guangzhou, China; 2 Shenzhen Pingle Orthopedic Hospital, (Shenzhen Pingshan Traditional Chinese Medicine Hospital), Shenzhen, China

**Keywords:** baicalein, gouty arthritis, inflammation, macrophage polarization, NF-κB signaling pathway, oxidative stress

## Abstract

**Objective:**

Gouty arthritis (GA) is an inflammatory disease caused by abnormal uric acid metabolism, with its pathological mechanism involving inflammatory cell infiltration and abnormal expression of pro-inflammatory factors. Monosodium urate (MSU) crystals activate the NLRP3 inflammasome, promoting the abnormal release of pro-inflammatory cytokines such as interleukin-1β (IL-1β), tumor necrosis factor-α (*TNF-α*), and interleukin-6 (IL-6), as well as the expression of mediators like monocyte chemoattractant protein-1 (MCP-1) and high mobility group box 1 (HMGB1), thereby amplifying local inflammatory responses. Additionally, MSU crystals activate Toll-like receptors (TLRs) and their downstream signaling pathways, including nuclear factor-κB (NF-κB) and mitogen-activated protein kinases (MAPK), driving the aggregation of inflammatory cells such as neutrophils and macrophages into the joint cavity, mediating synovial tissue damage, and causing oxidative stress imbalance. This study, based on the traditional Chinese medicine (TCM) theory of “accumulated toxins,” investigates the therapeutic effects and molecular mechanisms of the Chinese herbal monomer baicalein on gouty arthritis in mice through both *in vivo* and *in vitro* experiments.

**Methods:**

(1) *In Vivo* Experiments: Thirty BALB/c male mice were randomly divided into five groups: Control, Model, low-dose baicalein (Low,50 mg/kg), medium-dose baicalein (Middle,100 mg/kg), and high-dose baicalein (High,200 mg/kg). A mouse model of gouty arthritis was induced by intra-articular injection of MSU crystals. Behavioral scoring, gait analysis, joint swelling measurement, and histopathological analysis were used to evaluate the anti-inflammatory effects of baicalein. Molecular biology techniques were employed to detect serum and joint tissue levels of inflammatory cytokines (IL-1β, *TNF-α*, *iNOS*), immune regulatory markers (CD86, CD206). (2) *In Vitro* Experiments: RAW264.7 macrophages were cultured and divided into blank control, model (RAW264.7 cells treated with LPS 0.1 mg/ml and MSU 0.7 μmol/L), low-dose baicalein intervention (Low,10 μmol/L), medium-dose baicalein intervention (Middle,20 μmol/L), and high-dose baicalein intervention (High,30 μmol/L) groups. Real-time fluorescence quantitative PCR (RT-qPCR) was used to detect mRNA expression levels of IL-1β, *TNF-α*, *iNOS*, CD86, CD206 and IL-10. Cell proliferation was assessed using the CCK-8 assay. Protein expression levels of *TNF-α* and *iNOS* were determined by Western blotting. Immunofluorescence was used to observe the effect of baicalein on M1-type polarization of RAW264.7 cells. Cell migration was evaluated through a cell migration assay.

**Results:**

Baicalein intervention significantly alleviated joint swelling and inflammatory infiltration in a dose-dependent manner *(P* < 0.05). Molecular studies revealed that baicalein inhibited *iNOS*-mediated oxidative stress, downregulated the activation of the NF-κB signaling pathway, reduced the secretion of pro-inflammatory cytokines (IL-1β, *TNF-α*), and suppressed *iNOS* expression. Furthermore, baicalein regulated the expression of surface markers CD86 and CD206, thereby inhibiting M1 polarization and promoting the transition from an M1 to an M2 phenotype.

**Conclusion:**

This study demonstrates that baicalein improves the inflammatory microenvironment of gouty arthritis through multi-target and multi-pathway synergistic effects, including the inhibition of oxidative stress, immune regulation, and modulation of key signaling pathways. These findings provide experimental evidence supporting the clinical application of baicalein in the treatment of gouty arthritis.

## 1 Introduction

Gouty arthritis (GA) is a metabolic inflammatory disease caused by the deposition of urate crystals (primarily monosodium urate crystals, MSU) in joints and surrounding tissues, stemming from impaired uric acid metabolism (1, 2). The pathological mechanism primarily involves hyperuricemia induced by the overproduction or underexcretion of uric acid, which subsequently triggers the deposition of urate crystals within the joints. Clinically, GA manifests as acute articular erythema, swelling, heat, pain, functional impairment, and chronic joint deformities (3, 4). Driven by dietary shifts and the high prevalence of metabolic syndrome, the incidence of GA has been rising annually, making it the second most common metabolic disease after diabetes mellitus (5, 6). The Global Burden of Disease (GBD) study further shows that gout continues to increase in the global health burden of non-communicable diseases, especially among older men (7). A meta-analysis of national data from 2000 to 2014 showed that the prevalence of gout among adults in China was about 1%-3%, and hyperuricemia, as its key biochemical basis, was as high as about 14.0%.There are significant gender and age differences: the prevalence of the disease is much higher in men (about 24.5%) than in women (about 3.6%) (8). In terms of age distribution, the peak of male prevalence appears in young adults (18–39 years old), while the prevalence of female increases gradually with age, especially after menopause, and reaches the peak in old age. In addition, gout incidence shows a younger trend, which is closely related to changes in dietary patterns, increased alcohol intake and increased obesity rates. Hyperuricemia and gout not only cause joint damage, but also are associated with increased risk of systemic complications such as cardiovascular disease and chronic kidney disease, highlighting the urgency of comprehensive prevention and control (9). Therefore, a thorough understanding of its epidemiological characteristics and risk factors is of great significance for formulating targeted prevention and control strategies and reducing the overall disease burden.

**Figure 1:**
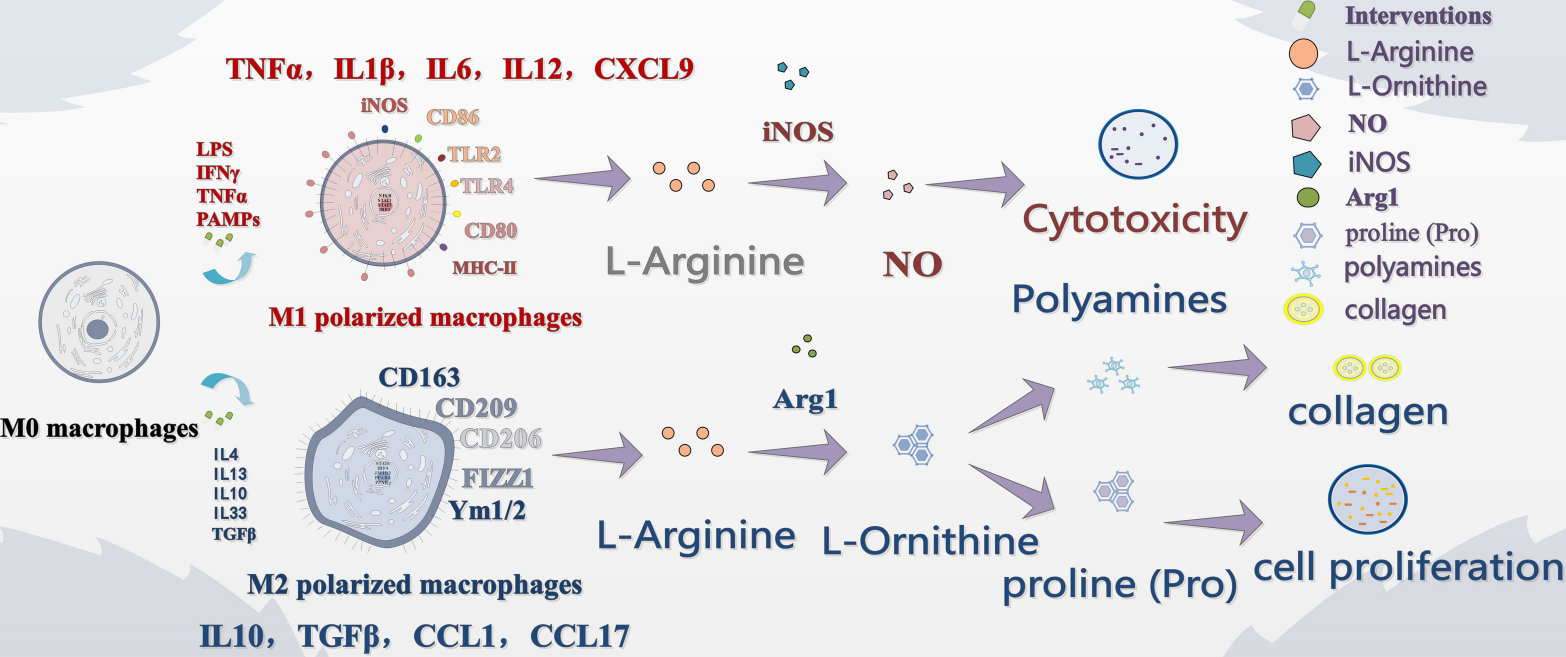
Schematic representation of macrophage polarization and associated metabolic and signaling pathways in the context of inflammatory responses.

MSU crystal deposition is the initial factor of GA in pathogenesis. Groundbreaking studies have shown that MSU crystals activate NLRP3 inflammatory bodies through lysosomal disruption, resulting in caspase-1 activation and IL-1β maturation release, which are central molecular events in gout inflammation (10). This process occurs primarily in macrophages within the joint cavity. Macrophages exhibit distinct polarization patterns during inflammatory responses: M0 macrophages differentiate into pro-inflammatory M1 phenotypes upon stimulation with LPS and Th1 cytokines (e.g., IFN-γ and TNF-α), whereas they polarize into anti-inflammatory M2 phenotypes under the influence of IL-4, IL-13, IL-10, IL-33, and TGF-β (11–18). Key studies have shown that MSU crystals are not only activators of NLRP3, but also strongly drive macrophage polarization toward the pro-inflammatory M1 phenotype. For example, studies have revealed that MSU induces macrophages to produce M1-related chemokines, recruiting neutrophils to trigger inflammation; uric acid itself can also “prime” human monocytes via the NLRP3 pathway, predisposing them to produce M1-type responses. (19, 20). High-level studies have further clarified that macrophage M1 polarization driven by NLRP3 inflammasome activation is a central event in the pathogenesis of gouty arthritis. This process not only triggers the explosion of key pro-inflammatory factors such as IL-1β, but also the accompanying reprogramming of cell metabolism (such as conversion to glycolysis), which is a key link in continuously amplifying inflammatory signals, leading to “inflammatory factor storm” and eventual joint tissue destruction (21, 22). Importantly, macrophage M2 polarization has been shown to play a protective role in inflammatory diseases by suppressing excessive inflammation and facilitating tissue healing (23, 24). In the context of gouty arthritis, promoting M2 polarization can counteract the pro-inflammatory effects of M1 macrophages, reduce synovial inflammation, and mitigate joint damage through enhanced anti-inflammatory cytokine production (25, 26) (**Figure 1**).

Currently, the pharmacological treatment of GA is limited by issues such as hepatorenal toxicity and high recurrence rates (27, 28), In contrast, Traditional Chinese Medicine (TCM) has demonstrated unique advantages in the management of GA. Research indicates that the multi-component and multi-target characteristics of TCM formulas and monomers can synergistically regulate purine metabolism, inhibit inflammation, and improve the microenvironment (21). Previous studies have confirmed that TCM monomers such as berberine and quercetin alleviate joint inflammation by inhibiting signaling pathways such as NF-κB and MAPK, thereby reducing the expression of pro-inflammatory cytokines like *TNF-α* and IL-1β (29–31). Building upon our team’s previous research, this study focuses on the TCM monomer baicalein. Based on the “Toxin Accumulation” (Xu Du) theory and utilizing both *in vivo* and *in vitro* experiments, we systematically elucidate its mechanism in alleviating synovial inflammation and joint damage by inhibiting NLRP3-mediated M1 polarization in MSU-activated synovial macrophages and suppressing the release of active IL-1β and *TNF-α* via the inflammasome signaling pathway (32, 33), Furthermore, the study investigates whether baicalein exhibits dose-dependent “bidirectional regulation” properties and synergistic mechanisms in combination therapies. This research aims to provide novel targets for the precision treatment of GA and to promote the transformation of modern TCM research from “component analysis” to “mechanism-application” integration, possessing both theoretical innovation and clinical translational value.

## 2 Materials and methods

### 2.1 Materials and reagents

#### 2.1.1 Animals and cell lines

A total of 30 healthy adult male SPF-grade Balb/c mice (7–8 weeks old, initial weight: 20 ± 2 g) were used in this study. All mice were procured from the Experimental Animal Center of Guangzhou University of Chinese Medicine and housed in an SPF-grade laboratory under standard conditions: temperature maintained at 22–25 °C, relative humidity of 50%–60%, and a 12-h light/dark cycle. Prior to the experiment, all animals underwent a 7-day acclimatization period with *ad* libitum access to standard chow and water. All animal procedures were strictly conducted in accordance with the ethical guidelines of the International Association for the Study of Pain and the Guide for the Care and Use of Laboratory Animals (2006) issued by the Ministry of Science and Technology of China. The study protocol was approved by the Animal Ethics Committee of Guangzhou University of Chinese Medicine (Approval No.: 20241010010). The institution holds valid licenses for laboratory animal production (SCXK (Yue) 2023-0068) and laboratory animal use (SYXK (Yue) 2023-0347). The mouse monocyte/macrophage cell line RAW264.7 (ATCC) was purchased from the American Type Culture Collection (ATCC) for *in vitro* experiments.

#### 2.1.2 Chemicals and drugs

Key reagents for inducing hyperuricemia included Potassium Oxonate and Yeast Extract (both from Shanghai Macklin Biochemical Co., Ltd.). Monosodium urate monohydrate crystals (MSU) for inducing acute gouty arthritis and Lipopolysaccharide (LPS, Sigma-Aldrich, Cat# L2880) were also procured. The test compound, Baicalein (purity ≥ 98%), was obtained from MedChemExpress (Cat# HY-N0152). Carboxymethyl cellulose sodium (CMC-Na), used as the vehicle for modeling and administration, was purchased from Shanghai Renjie Biotechnology Co., Ltd. Isoflurane, used as an anesthetic, was obtained from Shenzhen RWD Life Science Co., Ltd.

#### 2.1.3 Assay kits

Biochemical assay kits for serum uric acid (UA), serum creatinine (Scr), and blood urea nitrogen (BUN) were purchased from Nanjing Jiancheng Bioengineering Institute. Cell proliferation was assessed using the CCK-8 kit (Shanghai Beyotime Biotechnology). TRIzol^®^ Reagent (Thermo Fisher) was used for RNA extraction, and the PrimeScript™ RT Reagent Kit (Takara) was used for reverse transcription. SYBR Green Master Mix (Applied Biosystems) was utilized for real-time quantitative PCR (RT-qPCR). Protein concentration was determined using the BCA Protein Assay Kit (Shanghai Beyotime Biotechnology), and ECL chemiluminescent substrate (Thermo Fisher) was used for Western blot development.

#### 2.1.4 Antibodies

Primary antibodies for Western blot and immunofluorescence included: *TNF-α* (Proteintech, 60291-1-Ig), *iNOS* (Cell Signaling Technology, 13120), NLRP3 (Sigma-Aldrich, 29125), Caspase-1 (Sigma-Aldrich, 32096), and β-actin (Affinity, AF7018). Corresponding HRP-conjugated or fluorophore-conjugated secondary antibodies were purchased from Abcam and Thermo Fisher.

#### 2.1.5 Cell culture and related reagents

RAW264.7 cells were cultured in high-glucose DMEM (Gibco) supplemented with 10% fetal bovine serum (FBS, Gibco) and 1% penicillin-streptomycin solution (Guangzhou Jingxin Biotechnology). Cells were digested using 0.25% trypsin containing EDTA. For immunofluorescence, 4% paraformaldehyde, Triton X-100, and DAPI (all from Servicebio) were used for fixation, permeabilization, and nuclear staining, respectively.

#### 2.1.6 Instruments and equipment

Major instruments used in this study included: a Thermo Fisher Vanquish UHPLC system coupled with an Orbitrap Exploris 120 high-resolution mass spectrometer for compound analysis; an Applied Biosystems QuantStudio 5 Real-Time PCR System for gene expression analysis; a Bio-Rad ChemiDoc MP Imaging System for protein expression analysis; a Thermo Fisher Multiskan GO Microplate Spectrophotometer for luminometric assays; and a Leica DMI3000B Inverted Fluorescence Microscope for cell morphology observation. Tissue processing was performed using a Leica ASP300S Automatic Tissue Processor and an RM2235 Rotary Microtome. Centrifugation was conducted using an Eppendorf 5810R High-Speed Refrigerated Centrifuge. Cell manipulations were carried out in a *P* < 0.05 Qingdao Haier HR1200-IIA2 Clean Bench, with cells incubated in a Thermo Fisher Heracell 150i CO2 Incubator. MSU crystals and modeling suspensions were prepared using a Shanghai Shengxi SX-1000 Ultrasonic Dispenser.

### 2.2 *In vitro* experiments

**Figure 2:**
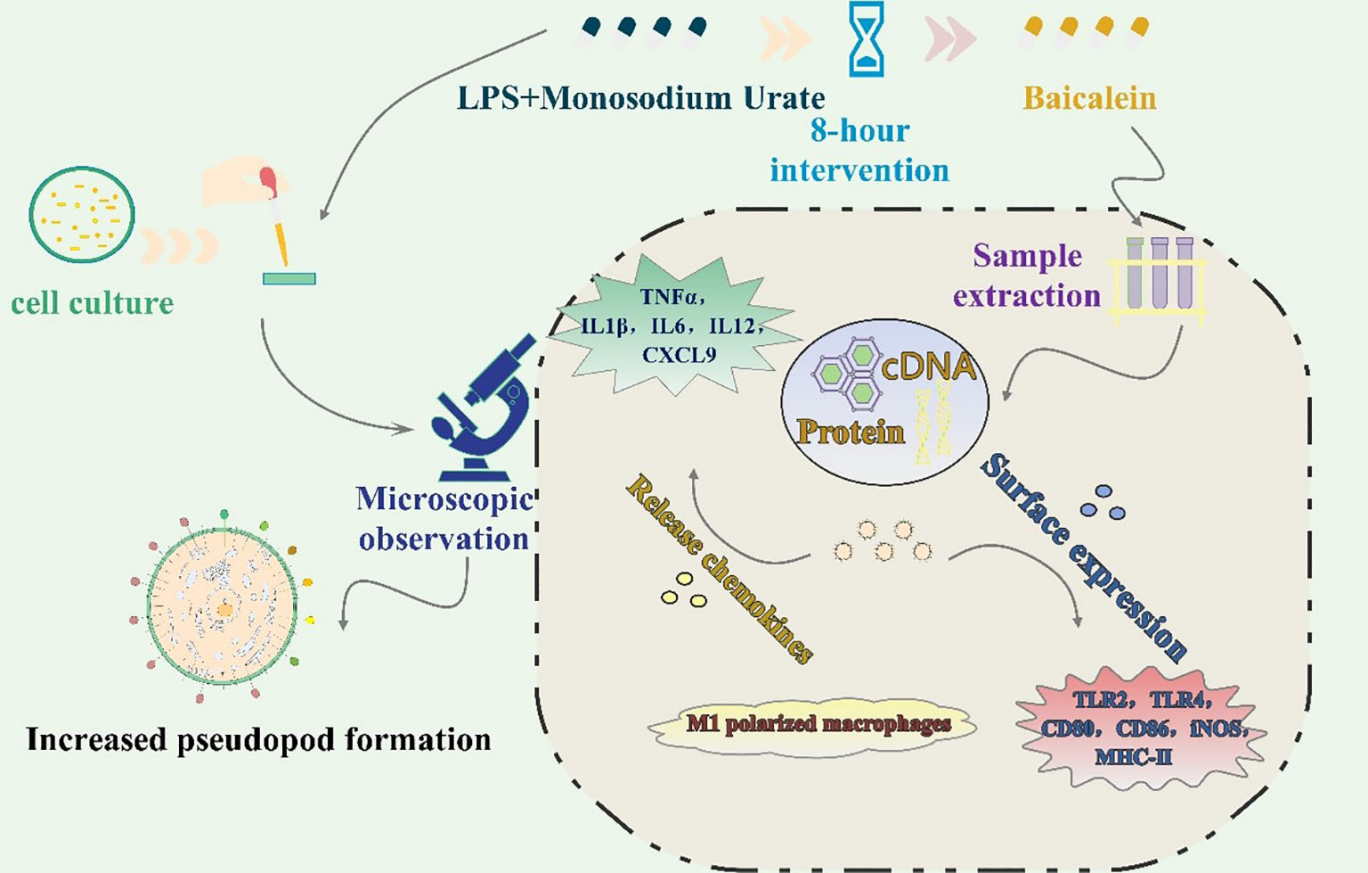
Schematic diagram of *in vitro* experiment.

This study aimed to evaluate the anti-inflammatory effects of baicalein on M1 polarization of RAW264.7 macrophages induced by lipopolysaccharide (LPS) and monosodium urate (MSU). Murine RAW264.7 macrophages were cultured under standard conditions and co-stimulated with LPS and MSU crystals to induce classical M1 polarization, simulating the inflammatory microenvironment of gouty arthritis (34). Baicalein was added 8 h post-stimulation as an intervention agent to assess its regulatory effects. Cellular responses were evaluated using multiple approaches: (1) Microscopic observation revealed increased pseudopod formation in activated macrophages, indicating enhanced migration and phagocytic activity (35);(2) Flow cytometry was employed to analyze the surface expression of M1 and M2 markers, including CD86 and CD206. Immunofluorescence assays were conducted to detect the expression of CD86, CD206, *iNOS*, and *TNF-α*; (3) Quantitative real-time PCR (qRT-PCR) was used to quantify pro-inflammatory cytokines such as *TNF-α*, IL-6, and CXCL9 to evaluate the secretion profile; (4) Total protein was extracted from treated cells for molecular analysis, including Western blotting. These methods allowed for a comprehensive assessment of baicalein’s capacity to inhibit M1 macrophage polarization and alleviate inflammation in a gout-related cellular model (**Figure 2**).

#### 2.2.1 Cell culture

RAW264.7 cells were routinely cultured in high-glucose DMEM supplemented with 10% (v/v) fetal bovine serum (FBS) and a penicillin-streptomycin antibiotic solution. Cells were maintained in a humidified incubator at 37 °C with 5% CO_2_. The culture medium was refreshed every 1–2 days, and cells were subcultured when confluence reached 80%–90%.

#### 2.2.2 Cell viability assay

The CCK-8 assay was utilized to assess the effects of different compounds on RAW264.7 cell proliferation and cytotoxicity. RAW264.7 cells were digested, seeded into 96-well plates at a density of 3 × 10³ cells/well, and incubated overnight to allow for adhesion. To simulate a hyperuricemic environment, a 43 mM MSU stock solution was diluted in complete DMEM to a final concentration of 0.7 umol/l and applied to the cells for 2 h. Subsequently, a 1 mM baicalein stock solution was diluted in complete DMEM to generate a concentration gradient (80, 70, 60, 50, 40, 30, 20, and 10 μmol/L) for intervention. The experimental groups were designated as follows: Blank group (medium only), Control group (cells + medium), and Baicalein intervention groups (cells + medium + varying concentrations of baicalein). Each group included four replicate wells (n=4). After 24 h of intervention, the original medium was discarded, and 10 μL of CCK-8 reagent was added to each well, followed by incubation at 37 °C in the dark for 2 h. The optical density (OD) at 450 nm was measured using a microplate reader. Cell viability (%) was calculated using the following formula:


$$Cell\;Viability\left(\%\right)=\left(OD_{treatment}-OD_{blank}\right)/\left(OD_{control}-OD_{blank}\right)\times 100\%$$

#### 2.2.3 Quantitative real-time PCR

RAW264.7 cells were induced and cultured for 5 days. The culture medium was aspirated, and cells were washed three times with PBS. Total RNA was extracted using the TRIzol reagent, and cDNA was synthesized using the PrimeScript RT Reagent Kit according to the manufacturer’s instructions. The PCR reaction system (10 μL total volume) consisted of 5 μL of 2× SYBR Green Premix, 0.4 μL of forward and reverse primers, 2.2 μL of RNase-free water, and 2 μL of cDNA template. The amplification protocol was as follows: initial denaturation at 95 °C for 10 min, followed by 40 cycles of denaturation at 95 °C for 20 s and annealing/extension at 60 °C for 30 s. *GAPDH* was used as the housekeeping gene, and the relative expression of inflammatory genes was calculated using the 2⁻ΔΔCt method. The primer sequences are listed in **Table 1**.

**Table 1:** Primer sequences used for qPCR.

Gene name	GenBank accession No.	Primer sequence (5’ → 3’)
*GapdhF*	NM_001289726.2	GCCTCCTCCAATTCAACCCT
*GapdhR*		CCCAATACGGCCAAATCCGT
*IL-1βF*	NM_008361.4	TGCCACCTTTTGACAGTGATG
*IL-1βR*		AAGGTCCACGGGAAAGACAC
*TNFαF*	NM_001278601.1	AGGCACTCCCCCAAAAGATG
*TNFαR*		CCACTTGGTGGTTTGTGAGTG
*iNOSF*	NM_001313921.1	TCTAGTGAAGCAAAGCCCAACA
*iNOSR*		TGATGGACCCCAAGCAAGAC
*CD86F*	NM_019388.3	ATGGACCCCAGATGCACCA
*CD86R*		TGTGCCCAAATAGTGCTCGT
*IL-6F*	NM_001314054.1	CAACGATGATGCACTTGCAGA
*IL-6R*		GTGACTCCAGCTTATCTCTTGGT
*CD206F*	NM_008625.2	TTCAGCTATTGGACGCGAGG
*CD206R*		GAATCTGACACCCAGCGGAA
*IL-10F*	NM_010548.2	GGTTGCCAAGCCTTATCGGA
*IL-10R*		CACCTTGGTCTTGGAGCTTATT
*CXCL10F*	NM_021274.2	CCACGTGTTGAGATCATTGCC
*CXCL10R*		GAGGCTCTCTGCTGTCCATC

#### 2.2.4 Western blot analysis

RAW264.7 cells were grouped and treated as described in Section 1.2.3. Following treatment, the culture medium was discarded, and cells were washed twice with pre-chilled PBS. RIPA lysis buffer containing protease and phosphatase inhibitors was added to each well, and cells were lysed on ice for 30 minutes. Cell lysates were harvested using a cell scraper, transferred to pre-chilled centrifuge tubes, and centrifuged at 12,000 × g for 15 minutes at 4 °C. The supernatant was collected, and protein concentration was determined using a BCA protein assay kit. Equal amounts of protein samples (typically 20–30 µg) were mixed with 5× loading buffer and denatured by boiling at 100 °C for 10 minutes. Proteins were separated by electrophoresis on 10% or 12% SDS-PAGE gels and subsequently transferred to PVDF membranes using the wet transfer method. The membranes were blocked with 5% non-fat milk in TBST at room temperature for 1 h. After blocking, the membranes were incubated with primary antibodies (*TNF-α*, 1:1000; *iNOS*, 1:1000; β-actin, 1:5000) diluted in blocking buffer overnight at 4 °C. The following day, membranes were washed three times with TBST for 10 minutes each, followed by incubation with HRP-conjugated secondary antibodies (1:5000) at room temperature for 1 h. After additional washes with TBST, protein bands were visualized using ECL chemiluminescent substrate and captured using a chemiluminescence imaging system. Band intensities were quantified using ImageJ software and normalized to β-actin as the internal reference.

#### 2.2.5 Flow cytometry

To detect surface markers of macrophage polarization, flow cytometry was employed. RAW264.7 cells were seeded in 6-well plates and treated according to the groups described in Section 1.2.3. After intervention, cells were digested with EDTA-free trypsin, and the cell suspension was collected and washed twice with pre-chilled PBS. Cells were resuspended in 100 µL of PBS and incubated with fluorescently labeled anti-mouse CD86-FITC and CD206-PE antibodies (or isotype control antibodies). The incubation was performed at 4 °C for 30 minutes, with strict protection from light throughout the entire process. Following incubation, cells were washed twice with PBS, resuspended in 300 µL of PBS, and immediately analyzed. Data were acquired using a flow cytometer and analyzed using FlowJo software. The gating strategy was as follows: First, cell debris and aggregates were excluded based on the forward scatter (FSC-H) and side scatter (SSC-H) distributions. Subsequently, CD11b+ cells were isolated from the total cell population and defined as the target macrophage population. Finally, within the CD11b+ gate, the proportions of CD86+ (M1-type) and CD206+ (M2-type) cells were calculated separately using quadrant percentage analysis.

#### 2.2.6 Wound healing (scratch) assay

RAW264.7 cells were seeded in 6-well plates at a density of 5 × 10^5^ cells/well and cultured in DMEM containing 10% FBS until confluence reached over 90%. A straight line was scratched vertically across the monolayer using a 200 µL sterile pipette tip, and the wells were gently washed three times with PBS to remove detached cell debris. The medium was replaced with 1% FBS-containing medium, and cells were treated with corresponding concentrations of MSU crystals and baicalein as described in Section 1.2.3. Images were captured at 0 and 12 h post-scratching at the same location using an inverted microscope. The wound area was measured using ImageJ software, and the migration rate was calculated as follows: Migration Rate (%)= (Area _0h_−Area _12h_)/Area _0h_ ×100%.

#### 2.2.7 Immunofluorescence staining

RAW264.7 cells were seeded in confocal dishes and treated according to the groups described in Section 1.2.3. After treatment, the medium was discarded, and cells were washed three times with PBS. Cells were fixed with 4% paraformaldehyde at room temperature for 15 minutes and washed three times with PBS. Permeabilization was performed using 0.1% Triton X-100 for 10 minutes, followed by three PBS washes. Cells were blocked with 5% BSA at room temperature for 1 h. The blocking solution was removed, and primary antibodies (anti-CD86, 1:200; anti-CD206, 1:200; anti-*iNOS*, 1:200; anti-*TNF-α*, 1:200) diluted in 1% BSA were added for overnight incubation at 4 °C. The next day, cells were washed three times with PBS and incubated with the corresponding fluorescent secondary antibody (Alexa Fluor 488-conjugated goat anti-rabbit IgG, 1:500) and DAPI (1:1000) at room temperature in the dark for 1 h. After three final washes with PBS, images were captured using a confocal microscope. Fluorescence intensity was analyzed using ImageJ software.

### 2.3 *In vivo* experiments

#### 2.3.1 Animal grouping and model establishment

**Figure 3:**
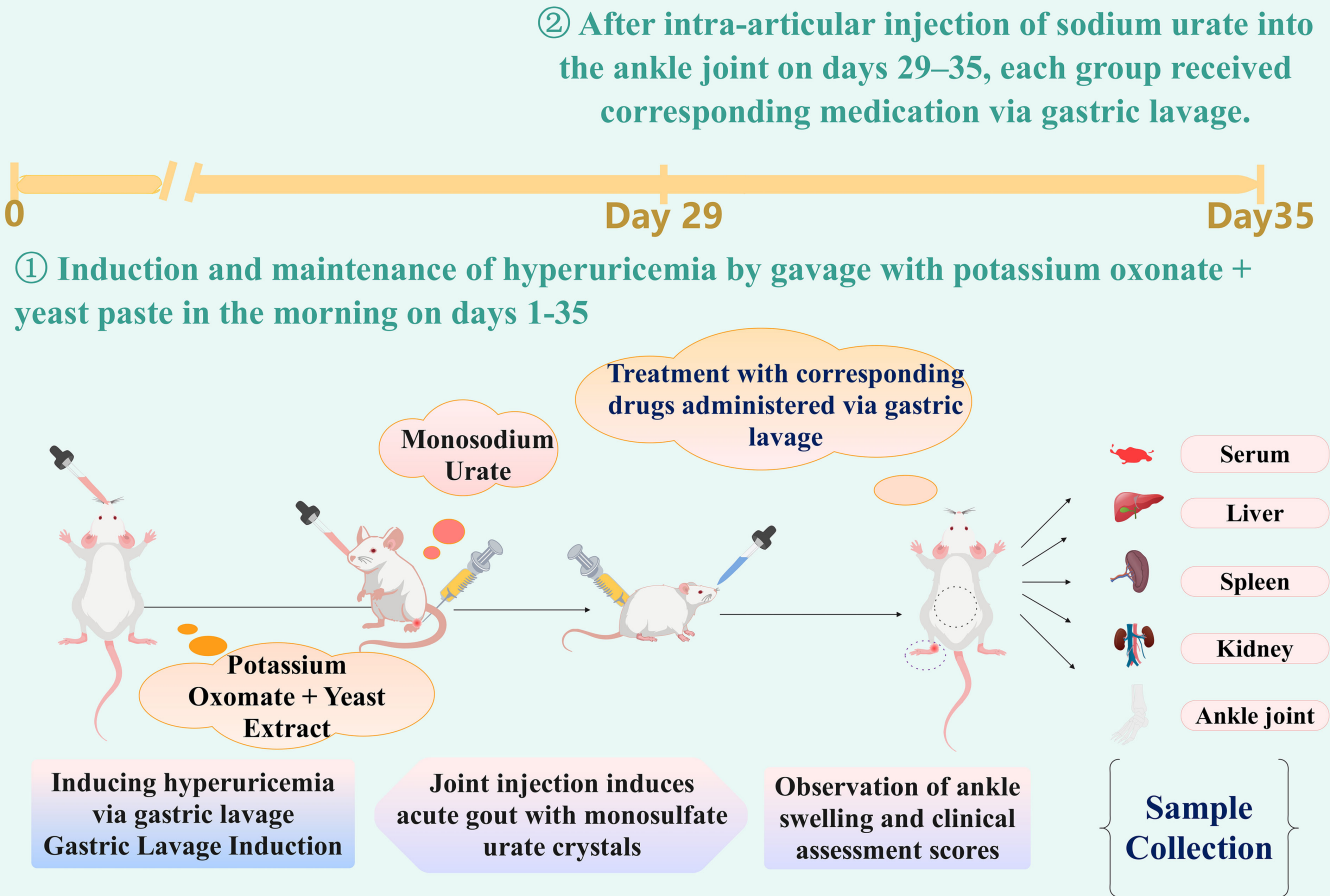
Schematic diagram of the experimental protocol for establishing a hyperuricemia-induced acute gouty arthritis mouse model and baicalein intervention. Male Balb/c mice were administered Potassium Oxonate (PO) and Yeast Extract via oral gavage from Day 1 to Day 35. Acute gouty arthritis was induced by intra-articular injection of monosodium urate (MSU) crystals into the left ankle joint from Day 29 to Day 35 (43). Starting from Day 29, all groups received daily oral gavage treatments (including baicalein and saline control). Relevant signs in mice, including body weight, food intake, water intake, urine output, ankle swelling, and gait, were monitored regularly. On Day 35, mice were euthanized, and serum, liver, kidney, and ankle joint tissues were collected for subsequent biochemical, histopathological, and molecular biological analyses. This protocol allows for a comprehensive evaluation of the anti-inflammatory and uricosuric effects of baicalein in a clinically relevant gout model.

Thirty healthy adult male SPF-grade Balb/c mice (7–8 weeks old, 20–22 g) were acclimatized in an SPF environment for one week and then randomly divided into 5 groups (n = 6) based on body weight: Control group (Control), Model group (Model), Baicalein low-dose group (Low, 50 mg/kg), Baicalein medium-dose group (Middle, 100 mg/kg), and Baicalein high-dose group (High, 200 mg/kg). To simulate the clinical onset of acute gout, a composite model was employed. Except for the Control group, which received 0.9% Sodium Chloride Solution via oral gavage every morning, the remaining groups were administered a hyperuricemia-inducing solution (containing Potassium Oxonate 500 mg/kg and Yeast Extract 10 g/kg) at a volume of 0.1 mL/10 g body weight daily for 35 consecutive days to induce and maintain hyperuricemia (36). On Day 29 of modeling, the acute gouty arthritis model was established according to literature methods: mice were anesthetized with isoflurane, and a puncture was made between the posterior protrusions on the lateral side of the left ankle joint. BD insulin needle was inserted into the joint cavity at a 45° angle until a characteristic ‘loss of resistance’ was perceived, confirming entry into the joint cavity. The Model group and Baicalein dose groups received an intra-articular injection of 50 µL of MSU crystal sterile solution (50 mg/mL); successful injection was confirmed by the bulging of the joint capsule on the contralateral side. The Control group received an equal volume of sterile saline. Oral gavage treatment commenced on the afternoon of the modeling day. The Control group received 0.9% Sodium Chloride Solution. The Model group received 0.5% CMC-Na solution, while the Baicalein groups received corresponding concentrations of baicalein suspension (dissolved in 0.5% CMC-Na) once daily for 7 consecutive days (37). On Day 35, after isoflurane anesthetized mice completed blood collection from the retro-orbital venous plexus, all mice were humanely euthanized to minimize pain and distress. Euthanasia was performed via an intraperitoneal injection of an overdose of sodium pentobarbital (150 mg/kg) (38). Death was confirmed by the permanent cessation of heartbeat and respiration, accompanied by the disappearance of the corneal reflex. Following euthanasia, synovial tissues and blood samples were collected immediately for subsequent histological and biochemical analyses. All animal procedures were approved by the Institutional Animal Care and Use Committee (**Figure 3**).

#### 2.3.2 Gait analysis

On Day 35, a mouse gait analysis system was used for data acquisition. Mice were placed in a transparent walking channel to walk freely, and a high-speed camera beneath the channel recorded plantar images. The analysis software automatically calculated and compared gait parameters, including Stance Phase, Stride Cycle, Bilateral Coordination, Max Contact Area, Interlimb Speed, and Swing Speed (39), to assess the degree of joint pain and functional impairment.

#### 2.3.3 Changes in body weight, water intake, and food intake

During the experimental period (Days 1–35), body weight was measured and recorded using an electronic balance at fixed times weekly for the first 4 weeks and daily for the 5th week. The water intake and feed consumption per cage were recorded, and the average daily water intake and food intake per mouse were calculated to evaluate the general state of the mice affected by the model and drugs.

#### 2.3.4 Measurement of ankle joint swelling

At fixed time points—before modeling (Day 28, as baseline), after modeling (Day 29, 4 h post-injection), and during the treatment period (Days 30, 31, 32, 33, 34, 35)—the circumference or diameter of the left ankle joint was measured using a digital vernier caliper.

#### 2.3.5 Detection of serum uric acid, creatinine, and urea nitrogen levels

Mice were anesthetized with isoflurane, and blood was collected from the retro-orbital venous plexus. After standing at room temperature for 30 minutes, blood was centrifuged at 3000 rpm for 15 minutes at 4 °C to separate the serum. Serum uric acid (UA), creatinine (Cr), and urea nitrogen (UN) levels were measured strictly according to the instructions of the respective detection kits using a multifunctional microplate reader to determine the absorbance at the specified wavelength. Concentrations were calculated based on the standard curve. Blood was collected weekly during the first 4 weeks and daily at a fixed time during the 5th week.

#### 2.3.6 ELISA detection of serum inflammatory cytokines

On Day 35, Isoflurane at a concentration of 3%-5% was inhaled by mice using oxygen as carrier gas at a flow rate of 0.5-1.0 L/min through induction boxes or masks until the mice entered anesthesia (40). After anesthesia was stabilized, isoflurane concentration was adjusted to 1%-2%, and oxygen supply was continued at the same flow rate to maintain the anesthesia state of mice until the blood collection operation was completed (41). According to the individual reaction and anesthesia depth of mice, the concentration and flow rate were adjusted appropriately, and the physiological indexes such as respiration and corneal reflex of mice were closely observed to ensure the anesthesia effect and animal safety. Blood was collected from the retro-orbital venous plexus. ELISA kits for mouse IL-1β and *TNF-α* were used, and the procedure was performed strictly according to the manufacturer’s instructions. Briefly: standards and samples were added to the wells of a pre-coated antibody plate, followed by incubation and washing. A biotinylated detection antibody was added, followed by another incubation and wash. Subsequently, horseradish peroxidase-conjugated streptavidin was added, followed by incubation and washing. The TMB substrate solution was added for color development, followed by the addition of a stop solution. The absorbance of each well was immediately measured at 450 nm using a microplate reader, and the serum concentrations of inflammatory cytokines were calculated based on the standard curve.

#### 2.3.7 Histopathological staining of ankle joint tissue

After sacrifice, the left ankle joint tissue was immediately excised and fixed in 4% paraformaldehyde for 48 h. Following fixation, decalcification was performed using 10% EDTA decalcification solution at 4 °C for approximately 2–3 weeks. After decalcification, tissues underwent conventional dehydration, clearing, and paraffin embedding. The tissue blocks were serially sectioned at a thickness of 5 µm using a rotary microtome. After deparaffinization and hydration, sections were stained with Hematoxylin and Eosin (H&E): nuclei were stained with hematoxylin, differentiated with hydrochloric acid alcohol, and cytoplasm was stained with eosin. After staining, sections were dehydrated, cleared, and mounted with neutral balsam. Under an optical microscope, synovial tissue inflammation cell infiltration, synovial hyperplasia, and cartilage destruction were observed, and histological scores were assessed.

## 3 Results

### 3.1 Baicalein inhibits LPS/MSU-induced pro-inflammatory cytokine mRNA expression in RAW264.7 cells

To evaluate the regulatory effect of baicalein on the inflammatory response of macrophages, we examined the mRNA expression levels of M1 polarization-related genes, including CXCL10, IL-1β, *TNF-α*, *iNOS*, CD86, and IL-6. The results demonstrated that stimulation with LPS combined with MSU significantly upregulated the expression of these genes (P < 0.0001). Specifically, IL-1β, *TNF-α*, *iNOS*, and CD86 were increased by approximately 6.2-fold, 5.8-fold, 4.1-fold, and 1.8-fold, respectively, compared to the control group. Pretreatment with baicalein significantly suppressed the expression of pro-inflammatory cytokines in a dose-dependent manner. In the high-dose group (80 μmol/L), the inhibition rates of IL-1β, *TNF-α*, and *iNOS* reached 82.3%, 81.6%, and 78.9%, respectively, which were significantly superior to those in the low-dose group (P < 0.001) (42). Furthermore, CD86 expression was also significantly downregulated (P < 0.001), indicating that baicalein effectively inhibits M1 macrophage polarization.

Simultaneously, the expression of M2 markers, CD206 and IL-10, was assessed. The results revealed that the mRNA levels of CD206 and IL-10 in the model group decreased by approximately 60% and 55%, respectively (P < 0.001), suggesting that inflammatory stimulation attenuates the anti-inflammatory phenotype. Following baicalein intervention, the expression of CD206 and IL-10 significantly recovered. In the high-dose group, levels returned to near-control levels (P < 0.01), with the relative expression of CD206 increasing from 0.42 ± 0.05 in the model group to 0.89 ± 0.07, and IL-10 increasing from 0.45 ± 0.06 to 0.91 ± 0.08 (P < 0.01). These findings indicate that baicalein not only inhibits M1 polarization but also promotes the conversion of macrophages to the M2 phenotype, thereby exerting a bidirectional regulatory role.

### 3.2 Baicalein reduces LPS/MSU-induced pro-inflammatory protein expression in RAW264.7 cells

**Figure 4:**
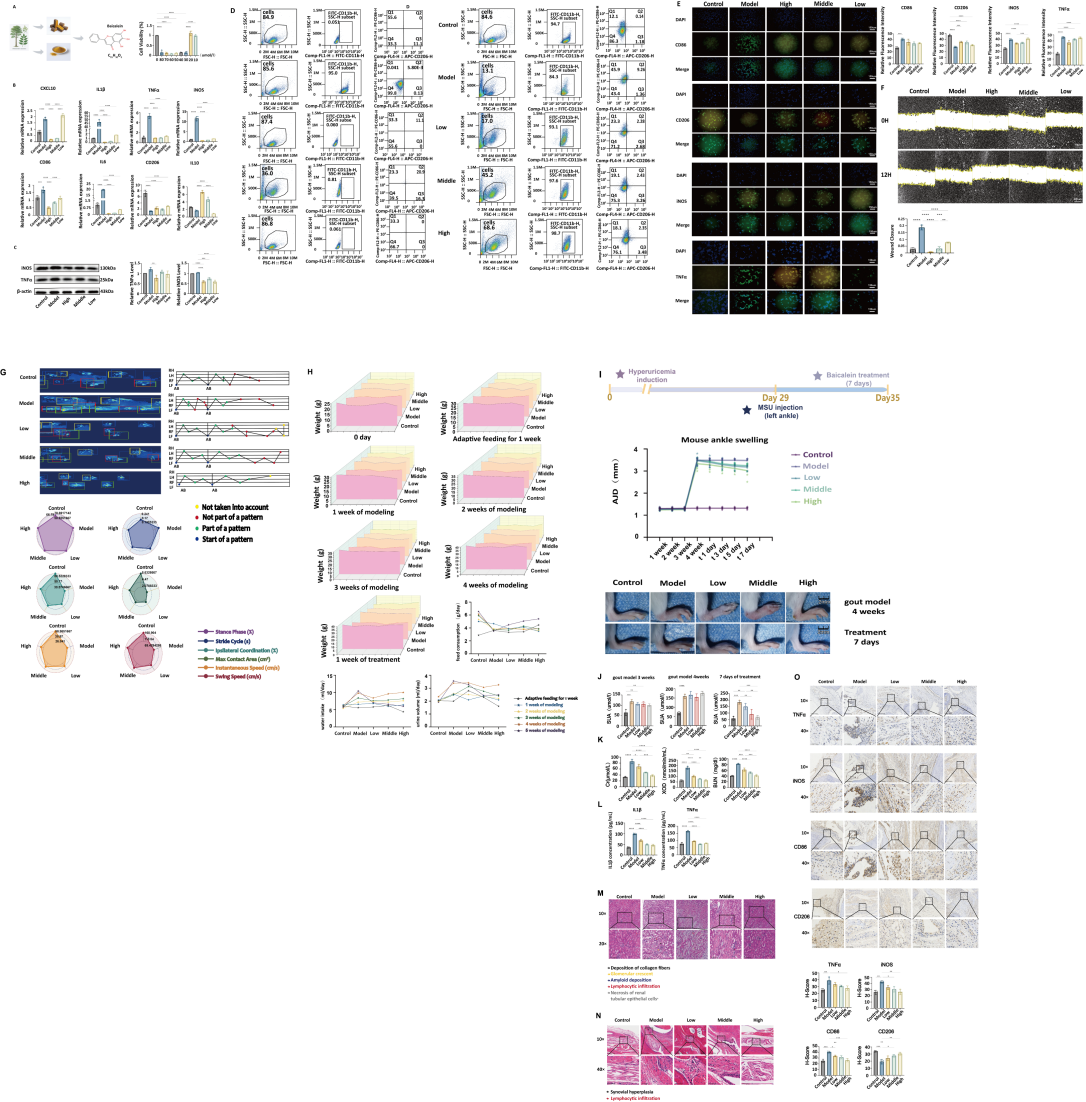
**(A)** Baicalein, derived from *Scutellaria baicalensis* (Huangqin), a key component of the “Dampness-Removing and Detoxifying” formula identified in our preliminary studies, has the molecular formula C_15_H_10_O_5_. **(B)** Effects of baicalein on the mRNA expression of M1/M2-related genes in RAW264.7 cells. The bar chart displays the relative expression levels of each group. *P < 0.05, **P < 0.01, ***P < 0.001 vs. Model group. M1 markers include CXCL10, IL-1β, *TNF-α*, *iNOS*, CD86, and IL-6; M2 markers include CD206 and IL-10. **(C)** Western blot analysis of the protein expression levels of inducible nitric oxide synthase (*iNOS*) and tumor necrosis factor-α (*TNF-α*), with β-actin serving as the internal reference. The bar graph presents the quantitative results of band intensity densitometry. Data are expressed as the mean ± standard deviation (SD) from three independent experiments. Statistical significance was determined by one-way analysis of variance (ANOVA) followed by Tukey’s *post-hoc* test: P < 0.01, P < 0.001, P < 0.0001 vs. Model group. **(D)** Flow cytometry analysis of the effects of baicalein on M1/M2 polarization in RAW264.7 cells. The left column displays Forward Scatter (FSC-H) versus Side Scatter (SSC-H) plots used for gating single viable cells; the middle column shows the CD11b^+^ macrophage population; the right column presents scatter plots of CD86 and CD206 expression within the CD11b^+^ cell population. Quadrant 1 (Q1) represents CD11b^+^/CD86^+^ cells (M1), and Quadrant 2 (Q2) represents CD11b^+^/CD206^+^ cells (M2). Data are presented as percentages. *P < 0.05, **P < 0.01, ***P < 0.001 vs. Model group. **(E)** Immunofluorescence staining was used to detect the effects of baicalein on the expression of M1/M2 markers in RAW264.7 cells. Upper panel: Fluorescence images showing DAPI (blue) staining for nuclei, while CD86, CD206, *iNOS*, and *TNF-α* are labeled in green or red, respectively. The “Merge” column displays the multi-channel fluorescence overlay. Lower panel: Bar chart of relative fluorescence intensity. Data are presented as mean ± SD. *P < 0.05, **P < 0.01, ***P < 0.001 vs. Model group. Scale bar = 150 µm. **(F)** Scratch wound healing assay detecting the effect of baicalein on the migratory capacity of RAW264.7 cells. Upper panel: Microscopic images at 0 h and 12 h (×100 magnification), with yellow dashed lines indicating the initial scratch boundary. Lower panel: Bar chart of wound closure rates for each group. Data are presented as mean ± SD. ***P < 0.001 vs. Control group; ###P < 0.001 vs. Model group. Scale bar = 500 µm. **(G)** Effects of baicalein on gait function in mice with gouty arthritis. Upper panel: Footprint track plots (left) and trend graphs of major gait parameter changes (right). Lower panel: Radar plots of gait parameters for each group, including Stance Phase, Stride Cycle, Bilateral Coordination, Max Contact Area, Interlimb Speed, and Swing Speed. Data are presented as mean ± SD. *P < 0.05, **P < 0.01, ***P < 0.001 vs. Model group. **(H)** Effects of baicalein on body weight, food intake, and excretion behavior in mice with gouty arthritis. Upper panel: Trend graph of body weight changes in each group (n = 6 per group, mean ± SD). Lower panel: Time-course curves of water intake, food intake, and urine volume. Data are presented as mean ± SD. *P < 0.05, **P < 0.01 vs. Model group. **(I)** Photographs taken at t7 (Day 35) illustrate the visible reduction in redness and swelling in baicalein-treated mice compared to the Model group. Longitudinal monitoring of ankle joint diameter (AJD) throughout the experimental period. The timeline consists of: adaptive feeding (week1); hyperuricemia induction (week2–week3); and the acute inflammatory phase with concurrent baicalein treatment (Day 29–35,t1–t7). Data are presented as mean ± SD (n = 6 per group). Statistical significance was determined by Two-way ANOVA followed by Tukey’s *post-hoc* test. *P<0.05, ** P<0.01, *** P<0.001 vs. Model group. **(J–L)** Effects of baicalein on serum biochemical indices in mice with gouty arthritis. **(J)** Serum uric acid (SUA) levels; **(K)**: Serum creatinine (Cr), xanthine oxidase (XOD), and blood urea nitrogen (BUN) levels; **(L)** Serum IL-1β and *TNF-α* concentrations. Data are presented as mean ± SD, n = 6. *P < 0.05, **P < 0.01, ***P < 0.001 vs. Model group. **(M)** Effects of baicalein on renal histopathology in mice with gouty arthritis (H&E staining, magnification: 10× and 20×). Arrows indicate major pathological features: collagen fiber deposition, glomerular crescent formation, amyloid deposition, lymphocyte infiltration, and renal tubular epithelial cell necrosis (indicated by arrows). n = 6. **(N)** Effects of baicalein on ankle joint histopathology in mice with gouty arthritis (H&E staining, 10× and 40× magnification). Arrows indicate major pathological features: synovial hyperplasia and lymphocyte infiltration (↓). n = 6. **(O)** Effects of baicalein on inflammatory cytokines and macrophage polarization markers in ankle joint tissues. Upper panel: Immunohistochemical staining images of *TNF-α*, *iNOS*, CD86, and CD206 (10× and 40× magnification); brown signals indicate positive expression. Lower panel: Bar chart of H-Score quantitative analysis for each indicator (n = 6, mean ± SD). *P < 0.05, **P < 0.01, ***P < 0.001 vs. Model group.

Western blot analysis revealed that the protein expression of *iNOS* and *TNF-α* was significantly enhanced in the model group, showing 3.5-fold and 2.8-fold increases compared to the control group, respectively (P < 0.001). After baicalein intervention, the band intensities of *iNOS* and *TNF-α* were markedly attenuated. In the high-dose group, protein expression levels were reduced to 35.6% and 38.2% of those in the model group (P < 0.001), which was consistent with the qPCR results (**Figure 4C**). This further confirms that baicalein exerts its anti-inflammatory effect by inhibiting the synthesis of key inflammatory proteins.

### 3.3 Regulatory effect of baicalein on M1/M2 phenotypic polarization in RAW264.7 cells

Flow cytometry was employed to detect the expression ratios of CD86^+^ (M1 marker) and CD206^+^ (M2 marker) in CD11b^+^ cells, further elucidating the regulatory role of baicalein in macrophage polarization. (44–46). The results demonstrated that under LPS/MSU co-stimulation, the proportion of CD11b^+^/CD86^+^ double-positive cells in the Model group was significantly elevated to 39.6% (*P* < 0.001), whereas the proportion of CD11b^+^/CD206^+^ double-positive cells plummeted from 11.1% in the Control group to 1.96% (*P* < 0.001). These findings suggest that the inflammatory environment induces robust M1 polarization while suppressing M2 conversion.

Following baicalein intervention, all dose groups significantly reversed this trend. In the high-dose group (80 μmol/L), the proportion of M1 cells was reduced to 23.3% (*P* < 0.01), approaching control levels; concurrently, the proportion of M2 cells rebounded to 10.6% (*P* < 0.05). The medium and low-dose groups exhibited similar but less pronounced effects; notably, the low-dose group still exhibited a distinct M1 bias (33.5%), indicating a dose-dependent anti-inflammatory effect (**Figure 4D**).

These results are consistent with the gene expression trends observed via qPCR, further confirming that baicalein achieves bidirectional regulation of macrophage function by inhibiting M1 polarization and promoting M2 polarization.

### 3.4 Baicalein downregulates M1 marker protein expression and upregulates M2 marker protein expression in RAW264.7 cells

To further validate the effect of baicalein on macrophage phenotype, immunofluorescence was employed to detect the intracellular localization and expression levels of CD86, CD206, *iNOS*, and *TNF-α*. The results showed that following LPS/MSU stimulation, the green fluorescence signals of CD86 and *iNOS* in the Model group were significantly enhanced, exhibiting a diffuse or granular distribution, while CD206 fluorescence was almost undetectable, indicating a dominant M1 polarization state. Concurrently, *TNF-α* expression was markedly elevated, primarily localized within the cytoplasm (**Figure 4E**).

Following baicalein pretreatment, distinct fluorescence changes were observed across all dose groups. In the high-dose group, the fluorescence intensities of CD86 and *iNOS* were significantly attenuated, decreasing by 58% and 61%, respectively, compared to the Model group (P < 0.001). *TNF-α* fluorescence was also notably reduced (P < 0.001). Simultaneously, CD206 fluorescence significantly recovered in the high-dose group, with its relative fluorescence intensity restoring to 67% of the Control group level (P < 0.01), suggesting partial activation of the M2 phenotype. The medium and low-dose groups also exhibited improvements, though the effects were less pronounced than in the high-dose group, further supporting a dose-dependent characteristic (**Figure 4E**).

Quantitative analysis results were consistent with qPCR and Western blot data, confirming that baicalein regulates macrophage function by inhibiting the expression of M1-related proteins (CD86, *iNOS*, *TNF-α*) and promoting the expression of the M2 marker (CD206).

### 3.5 Baicalein inhibits LPS/MSU-induced migratory capacity of RAW264.7 cells

The scratch wound healing assay was performed to assess cell migratory capacity and investigate the effect of baicalein on macrophage chemotaxis. The results showed that at 0 h, the scratch width was consistent across all groups. After 12 h, the wound closure rate in the Model group significantly increased to 21.8%, representing a more than 3-fold increase compared to the Control group (5.2%, P < 0.001), indicating that LPS/MSU stimulation significantly enhances the migratory activity of RAW264.7 cells.

Following baicalein pretreatment, cell migration was markedly inhibited: the wound closure rate in the high-dose group decreased to 6.3%, showing a highly significant difference compared to the Model group (P < 0.001). The medium and low-dose groups also exhibited varying degrees of inhibition, with the low-dose group retaining some migratory capacity (13.2%). These results indicate that baicalein can alleviate local inflammatory responses by inhibiting the migratory capacity of macrophages, thereby reducing their accumulation at inflammatory sites (**Figure 4F**).

### 3.6 Baicalein improves gait dysfunction in mice with gouty arthritis

A dynamic gait analysis system was employed to evaluate the impact of baicalein on motor function in mice with gouty arthritis by analyzing their walking patterns. The results revealed distinct gait abnormalities in the Model group compared to the Control group, characterized by asymmetric footprints, hindlimb dragging, shortened stride length, and delayed leg lifting. Quantitative analysis of gait parameters demonstrated that the Model group exhibited a significantly shortened Stance Phase (P < 0.001), prolonged Stride Cycle, decreased Bilateral Coordination, reduced Max Contact Area, and significantly lower Swing Speed and walking speed (P < 0.01), indicating severe impairment of motor capacity due to joint pain.

Following baicalein intervention, gait patterns improved in a dose-dependent manner across all treatment groups. In the high-dose group (200 mg/kg), mouse footprints became more symmetric, and gait appeared smoother, with all parameters showing significant improvement compared to the Model group *(P* < 0.05–0.001). Specifically, the Stance Phase returned to normal levels, the Stride Cycle was shortened, bilateral coordination was enhanced, contact area increased, and swing speed and walking speed were markedly elevated. The medium and low-dose groups also showed a trend of improvement, though the effects were less pronounced than in the high-dose group. The radar plots further visualized that the high-dose group approached Control levels across all measured parameters, indicating that baicalein effectively alleviates motor dysfunction induced by joint inflammation (**Figure 4G**).

### 3.7 Baicalein improves general status and physiological parameters in mice with gouty arthritis

Throughout the experimental period, body weight, water intake, food intake, and urine volume were recorded weekly for the first 4 weeks and daily at the same time point during the 5th week. The results indicated that during the acclimatization phase, all groups exhibited stable weight gain with no significant differences. Upon entering the modeling phase, weight gain in the Model group stagnated, with a distinct downward trend emerging in the 3rd week. By the 4th week, body weight was approximately 12.6% lower than that of the Control group (P < 0.01), suggesting that chronic inflammation negatively impacts systemic metabolism. Following one week of baicalein intervention, the high-dose group exhibited a rapid recovery in body weight, approaching Control levels. The medium and low-dose groups also showed varying degrees of recovery, though the effects were less pronounced than in the high-dose group (**Figure 4H**).

Regarding water intake, the Model group displayed a significant increase starting from the 3rd week (rising from 8.4 mL/cage/day to 10.4 mL/cage/day, *P* < 0.05), indicative of polydipsia potentially associated with hyperuricemia. In contrast, the high-dose group maintained water intake within the normal range without a significant increase. In terms of food intake, the Model group showed reduced consumption starting from the 2nd week, reaching a nadir in the 4th week (17.59 g/cage/day), which was significantly lower than the Control group (32.7 g/cage/day, P < 0.01). The high-dose group restored food intake to 27.01 g/cage/day, significantly outperforming the Model group (*P* < 0.05), demonstrating that baicalein improves appetite. Concerning urine volume, the Model group exhibited a progressive increase with modeling progression, reaching 3.55 mL/cage/day in the 4th week, significantly higher than the Control group (1.916 mL/cage/day, P < 0.01), suggesting an increased renal excretory burden. The high-dose group effectively controlled urine volume, maintaining it at 2.482 mL/cage/day, close to Control levels.

These findings indicate that baicalein not only alleviates joint inflammation but also ameliorates systemic metabolic disturbances, mitigating clinical manifestations of gout such as polydipsia, hypophagia, and polyuria, thereby enhancing the overall health status of the animals (**Figure 4H**). In addition, gross anatomical observation showed that there was no obvious abnormality in liver tissue morphology of mice in each group, combined with the recovery of body weight and renal function indicators, indicating that high dose scutellarin had good systemic safety in this experimental cycle.

### 3.8 Baicalein significantly alleviates ankle swelling in mice with gouty arthritis

To simulate the clinical progression of gouty arthritis, we established a composite mouse model involving pre-existing hyperuricemia followed by acute Monosodium Urate (MSU) crystal-induced inflammation. The longitudinal changes in ankle joint diameter (AJD) were monitored throughout the experimental period (**Figure 4I**). During the initial phase (Weeks 2–3), mice in the Model and baicalein-treated groups exhibited a stable but slight increase in AJD (approximately1.30 mm to 1.35 mm) following the administration of yeast extract and potassium oxonate, reflecting the systemic impact of hyperuricemia induction. On Day 29 (t1), following the first intra-articular injection of MSU crystals, the Model group showed a dramatic inflammatory surge, with the AJD reaching 3.55 ± 0.05 mm, significantly higher than that of the Control group (1.32 ± 0.05 mm, *P < 0.001*). During the therapeutic phase (t1–t7), continuous MSU provocations maintained severe swelling in the Model group, which remained at 3.54 ± 0.05 mm by Day 35 (t7). Baicalein treatment demonstrated a significant dose- and time-dependent inhibitory effect on joint edema. Specifically, the High-dose baicalein group exhibited a rapid therapeutic response; the AJD significantly decreased to 2.23 ± 0.12 mm at t1 (*P < 0.001 vs. Model*) and further declined to 1.76 ± 0.09 mm by t7, effectively approaching the baseline level. The Middle-dose group showed a steady decline, with the AJD reaching 2.64 ± 0.15 mm at t7 (*P < 0.01 vs. Model*). In contrast, the Low-dose group exhibited a marginal reduction to 2.91 ± 0.11 mm at t7, which did not reach statistical significance compared to the Model group (*P > 0.05*) (**Table 2**). These quantitative findings were further corroborated by representative morphological images at t7 (**Figure 4I**), where baicalein, particularly at the high dose, markedly reduced erythema and joint thickening.

**Table 2:** Changes in Ankle Joint Diameter (AJD) across different experimental phases (mm, { $\bar{x}$} ± s).

	1week	2week	3week	4week	Treatment 1 day	Treatment 3 day	Treatment 5 day	Treatment 7 day
Control	1.30 ± 0.05	1.30 ± 0.05	1.31 ± 0.05	1.32 ± 0.05	1.32 ± 0.05	1.32 ± 0.05	1.32 ± 0.05	1.32 ± 0.05
Model	1.28 ± 0.04	1.28 ± 0.04	1.29 ± 0.04	3.49 ± 0.12	3.49 ± 0.12	3.49 ± 0.12	3.49 ± 0.12	3.48 ± 0.12
Low	1.27 ± 0.08	1.28 ± 0.07	1.29 ± 0.07	3.47 ± 0.22	3.39 ± 0.22	3.29 ± 0.20	3.22 ± 0.19	3.18 ± 0.19^*^
Middle	1.28 ± 0.05	1.28 ± 0.05	1.29 ± 0.05	3.50 ± 0.15	3.43 ± 0.14	3.33 ± 0.15	3.28 ± 0.14	3.24 ± 0.13^*^
High	1.24 ± 0.03	1.25 ± 0.03	1.25 ± 0.03	3.33 ± 0.22	3.28 ± 0.22	3.20 ± 0.20^*^	3.12 ± 0.20^*^	3.02 ± 0.31^*^

Statistical significance indicated by asterisks (*) represents P < 0.05 compared to the Model group.

### 3.9 Baicalein reduces serum uric acid levels and improves renal function

To evaluate the effects of baicalein on hyperuricemia and renal injury, we measured serum levels of uric acid (SUA), creatinine (Cr), xanthine oxidase (XOD), and blood urea nitrogen (BUN) in each group. The results showed that SUA levels in the Model group were significantly elevated during the 3rd and 4th weeks of modeling, reaching 100.2 ± 8.6 μmol/L and 148.5 ± 10.3 μmol/L, respectively, representing increases of approximately 67% and 147% compared to the Control group (60.1 ± 5.2 μmol/L, P < 0.001). Following 7 days of baicalein intervention, the high-dose group exhibited a significant reduction in SUA to 82.4 ± 6.8 μmol/L (P < 0.001 vs. Model), while the medium and low-dose groups also showed a downward trend to varying degrees.

Regarding renal function, the Model group displayed significantly elevated Cr and BUN levels (78.3 ± 7.1 μmol/L and 89.6 ± 6.4 mg/dL, respectively), indicating impaired glomerular filtration. In the high-dose group, Cr decreased to 45.2 ± 5.3 μmol/L (P < 0.001) and BUN to 60.1 ± 5.8 mg/dL (P < 0.01), suggesting a protective effect of baicalein on the kidneys. Furthermore, XOD activity was significantly enhanced in the Model group (205.4 ± 12.3 nmol/min/mL) but was effectively inhibited in the high-dose group to 102.6 ± 9.4 nmol/min/mL (P < 0.001), indicating that baicalein exerts its uric acid-lowering effect by inhibiting the key enzyme responsible for uric acid production (see **Figures 4J, K**).

### 3.10 Baicalein inhibits the release of pro-inflammatory cytokines IL-1β and *TNF-α* in serum

ELISA results revealed that serum concentrations of IL-1β and *TNF-α* in the Model group were significantly elevated, reaching 98.6 ± 8.2 pg/mL and 168.3 ± 10.1 pg/mL, respectively. This represented increases of nearly 3-fold and 5-fold compared to the Control group (25.4 ± 4.1 pg/mL and 32.7 ± 3.8 pg/mL, P < 0.001). Following baicalein treatment, the high-dose group showed a significant reduction in IL-1β and *TNF-α* levels to 31.2 ± 5.6 pg/mL and 62.4 ± 6.8 pg/mL, respectively (P < 0.001 vs. Model). The medium and low-dose groups also exhibited inhibitory effects, albeit to a lesser extent. These results indicate that baicalein effectively suppresses systemic inflammatory responses and reduces the release of key pro-inflammatory cytokines (see **Figure 4L**).

### 3.11 Baicalein alleviates renal tissue pathological damage induced by gouty arthritis

Kidney tissues from each group were subjected to H&E staining and microscopic examination to assess the protective effect of baicalein against gout-related renal injury. The Control group exhibited intact renal architecture with clear glomerular outlines and regular tubular arrangement, without significant inflammatory cell infiltration or fibrosis. In contrast, the Model group displayed typical pathological features of gouty nephropathy: marked mesangial proliferation and glomerular crescent formation within the glomeruli, accompanied by extensive amyloid deposition. The renal interstitium showed dense lymphocyte infiltration, with degeneration and necrosis of some renal tubular epithelial cells, alongside excessive collagen fiber deposition, indicative of chronic inflammation and fibrosis.

Following baicalein intervention, renal pathology improved in a dose-dependent manner. In the high-dose group, glomerular structure was largely restored, amyloid deposition was significantly reduced, inflammatory cell infiltration was markedly alleviated, necrotic foci in renal tubular epithelial cells disappeared, and collagen fiber deposition approached normal levels. The medium-dose group showed moderate repair, while the low-dose group exhibited limited improvement with still-visible pathological changes. These findings suggest that baicalein effectively mitigates renal parenchymal damage caused by gouty arthritis by inhibiting inflammatory responses and fibrosis (see **Figure 4M**).

### 3.12 Baicalein alleviates synovial inflammation in ankle joints induced by gouty arthritis

To evaluate the intervention effect of baicalein on local joint lesions, ankle joint tissues were stained with H&E and examined microscopically. The Control group showed normal synovial structure with a thin synovial layer, orderly cell arrangement, no significant inflammatory cell infiltration, and intact cartilage surfaces. The Model group exhibited typical pathological features of acute gouty arthritis: significant synovial hyperplasia, dense proliferation of cells in the subsynovial layer, massive lymphocyte infiltration, and local neutrophil aggregation, indicating a severe inflammatory response. Mild erosion was observed at the cartilage margins in some areas.

After baicalein intervention, synovial inflammation was alleviated to varying degrees across the treatment groups. In the high-dose group, synovial hyperplasia was significantly reduced, with thickness approaching normal levels, inflammatory cell infiltration was markedly decreased, and cartilage structure remained intact. The medium-dose group showed moderate improvement, while the low-dose group had limited improvement, with visible synovial thickening and inflammatory cell aggregation. These results demonstrate that baicalein effectively reduces local tissue damage caused by gouty arthritis by suppressing synovial inflammation (see **Figure 4N**).

### 3.13 Baicalein inhibits M1 macrophage activation and promotes M2 polarization in ankle joints

Immunohistochemical staining of ankle joint tissues was performed to detect the expression levels of the pro-inflammatory cytokines *TNF-α* and *iNOS*, as well as the macrophage surface markers CD86 (M1 phenotype) and CD206 (M2 phenotype). The Control group showed weak expression of *TNF-α*, *iNOS*, and CD86, with moderate expression of CD206, indicating a low baseline inflammatory state. The Model group exhibited distinct M1 polarization characteristics: widespread and strong positive expression of *TNF-α* and *iNOS* in synovial tissues, a significant increase in CD86-positive cells, and markedly weakened CD206 expression, indicating a pro-inflammatory shift in the microenvironment.

Following baicalein intervention, all treatment groups showed dose-dependent improvements. In the high-dose group, the expression of *TNF-α*, *iNOS*, and CD86 was significantly downregulated (H-Scores: 35.4 ± 3.2, 32.1 ± 2.8, and 30.6 ± 2.9, respectively), representing reductions of approximately 42%, 45%, and 48% compared to the Model group (P < 0.01). Concurrently, CD206 expression was significantly enhanced (H-Score: 36.7 ± 3.1), approaching or even exceeding Control levels (P < 0.001). The medium and low-dose groups also showed trending improvements, though the effects were less potent than in the high-dose group. These results indicate that baicalein effectively regulates the local inflammatory response in gouty arthritis by inhibiting M1 macrophage activation, reducing pro-inflammatory cytokine release, and promoting a shift toward the anti-inflammatory M2 phenotype (see **Figure 4O**).

### 3.14 Molecular docking simulation of baicalein

**Figure 5:**
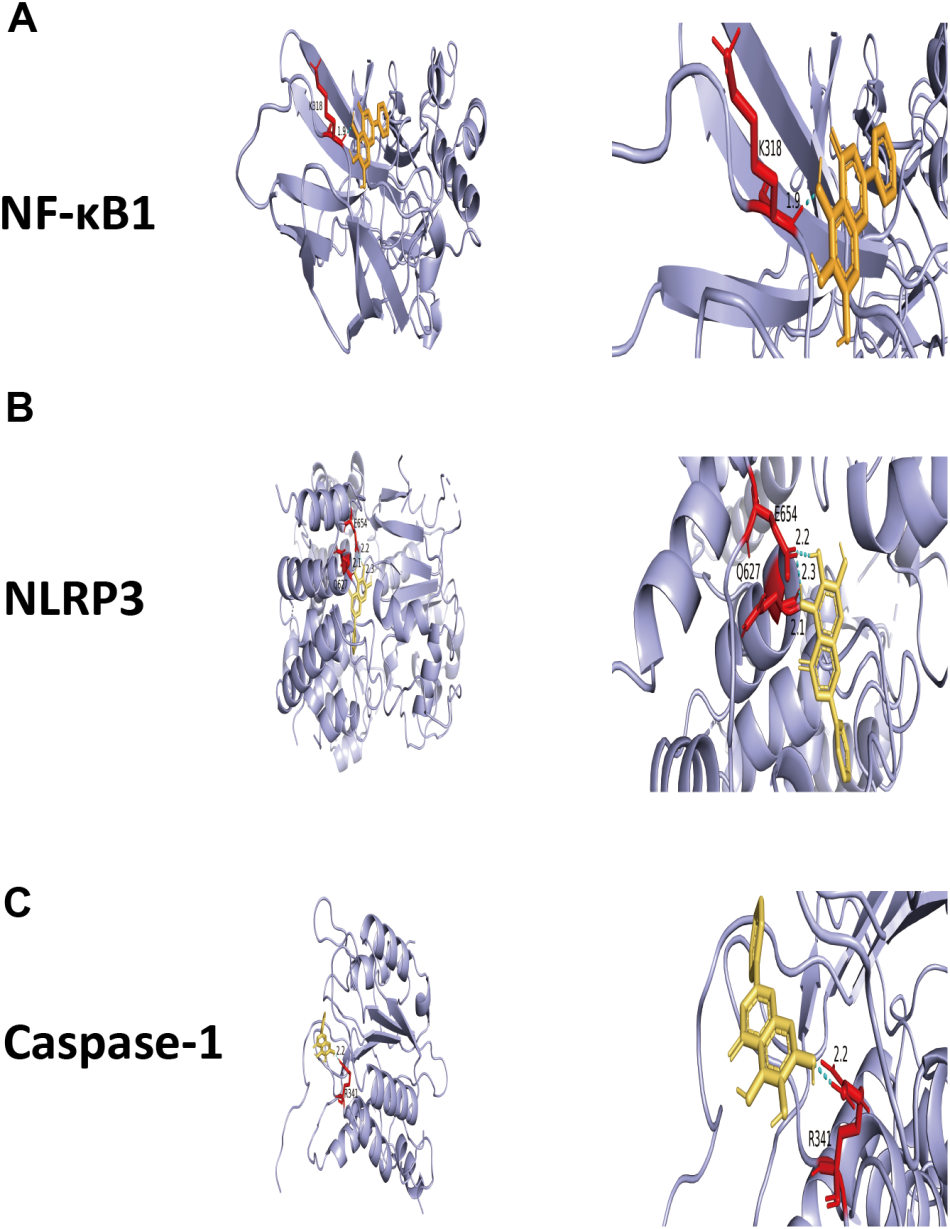
Molecular docking analysis of baicalein. Molecular docking of baicalein with core inflammatory proteins:NF-κB1: Baicalein binds to NF-κB1 with a minimum binding energy of *-4.04kcal/mol*. A hydrogen bond is formed between the ligand and the LYS318 residue at a distance of 1.9Å.NLRP3: The binding energy between baicalein and NLRP3 is -*6.16kcal/mol*. The interaction involves two hydrogen bonds with the GLU654 residue and one with GLN627, with bond distances of 2.2Å, 2.3Å, and 2.1Å respectively.Caspase-1: Baicalein exhibits molecular contact with the ARG341 residue of Caspase-1, forming a hydrogen bond with a distance of 2.2Å.

To further explore the potential direct molecular targets of baicalein in alleviating gouty arthritis, molecular docking simulations were performed to evaluate its binding affinity with core proteins of the NLRP3 inflammasome and NF-κB signaling pathways. The results demonstrated that baicalein exhibited a robust binding affinity for the NLRP3 protein, with a minimum binding energy of *-6.16kcal/mol*. Conformational analysis revealed that the baicalein molecule stably fits into the active binding pocket of NLRP3, forming three stable hydrogen bonds with residues GLU654 (E654) and GLN627 (Q627) at distances of 2.2Å, 2.3Å, and 2.1 Å, respectively. Regarding the NF-κB signaling pathway, baicalein showed significant binding potential with the NF-κB1 protein, yielding a minimum binding energy of -*4.04kcal/mol*. The three-dimensional docking model highlighted a strong hydrogen bond interaction between baicalein and the LYS318 (K318) residue with a distance of 1.9 Å. Additionally, the simulation identified specific molecular contact between baicalein and the Caspase-1 protein through a hydrogen bond with the ARG341 (R341) residue at a distance of 2.2Å. Collectively, these molecular docking results suggest that baicalein possesses the structural basis to directly intervene in both the “priming” stage (NF-κB1) and the “activation” stage (via NLRP3 and Caspase-1) of the inflammatory cascade, providing a robust theoretical foundation for the significant downregulation of IL-1*β*, TNF-*α*, and iNOS observed in our experimental findings (**Figure 5**).

## 4 Discussion

Baicalein precisely modulates macrophage polarization to reshape the inflammatory microenvironment in gouty arthritis, thereby elucidating its unique anti-inflammatory mechanism. MSU crystal-induced M1 polarization of macrophages is a key driver of the inflammatory response in gout (16, 47, 48), Our findings demonstrate that baicalein exerts a potent anti-inflammatory effect by orchestrating a phenotypic shift from the pro-inflammatory M1 state to the anti-inflammatory M2 state, thereby rebalancing the synovial microenvironment, which not only significantly inhibits the expression and release of pro-inflammatory cytokines such as IL-1β, *TNF-α*, and *iNOS* but also promotes the expression of anti-inflammatory and tissue repair factors like CD206 and IL-10. This precise modulation not only attenuates the continuous amplification of inflammatory signals but also facilitates the resolution of inflammation and the repair of joint tissues, offering a novel therapeutic strategy for gouty arthritis.

Further studies indicate that the anti-inflammatory effect of baicalein is closely associated with its inhibition of the NF-κB/*iNOS*-mediated oxidative stress response. The persistent activation of the NF-κB signaling pathway and the overexpression of *iNOS* are critical causes of oxidative stress imbalance and tissue damage in gouty arthritis (49–51). Baicalein significantly downregulates *iNOS* expression by specifically blocking NF-κB activation, thereby effectively inhibiting the excessive generation of NO and ROS and alleviating oxidative stress injury in joint tissues (52). Specifically, although direct phosphorylation of p65 was not presented in this study, our findings that baicalein significantly downregulated iNOS—a primary and essential downstream functional executor driven by NF-κB transcriptional activity—provide robust functional evidence of the suppressed signaling cascade. This inhibition effectively disrupts the amplification of inflammatory signals at the effector level. Furthermore, baicalein disrupts the vicious cycle of “inflammation-oxidative stress-polarization imbalance,” further enhancing its anti-inflammatory efficacy. This highlights the multi-target advantage of TCM monomers in the treatment of complex diseases. To further substantiate these multi-target effects, molecular docking was performed to simulate the direct interactions between baicalein and core inflammatory components. The results demonstrated that baicalein possesses high binding affinity for NLRP3 (binding energy:*-6.16 kcal/mol* and NF-κB1 (binding energy: *-4.04 kcal/mol*. Notably, baicalein formed stable hydrogen bonds with key residues such as GLU654 in NLRP3 and LYS318 in NF-κB1. Furthermore, the molecular contact between baicalein and Caspase-1 (hydrogen bond with ARG341) provides a theoretical basis for its ability to inhibit IL-1*β* maturation, even in the absence of direct protein expression data for Caspase-1 (53–54). These computational findings align with our experimental observations, confirming that baicalein intervenes in the inflammatory cascade by targeting both the ‘priming’ (NF-κB1) and ‘activation’ (NLRP3 inflammasome) stages (55–57).

This study not only provides solid experimental evidence for baicalein as a potential anti-gout drug but also demonstrates the alignment between the TCM theory of “Toxin Accumulation” and modern immunological mechanisms. Baicalein, derived from *Scutellaria baicalensis* (Huangqin), a TCM herb known for clearing heat and detoxifying, exerts its effects by regulating macrophage function through multiple pathways, inhibiting the release of inflammatory toxic signals, and promoting inflammation resolution. These mechanisms are highly consistent with the traditional TCM efficacy of “clearing heat and detoxifying, unblocking collaterals and relieving pain.” Future research should further explore the direct molecular targets of baicalein, its pharmacokinetics, and its potential for combination therapies, with the aim of developing more efficient and safer treatment regimens for gout and advancing the modernization of TCM research.

## Data Availability

The datasets presented in this study can be found in online repositories. The names of the repository/repositories and accession number(s) can be found in the article/**Supplementary Material**.
