# Supplementary material for: Baicalein links macrophage M2 polarization with reduced synovial inflammation to alleviate gouty arthritis
Source: Front Immunol. 2026 Apr 23;17:1812532. doi: 10.3389/fimmu.2026.1812532 (PMC13149162; doi:10.3389/fimmu.2026.1812532)
Supplement: Supplementary file 1 [file SupplementaryFile1.pdf]

frontiers

The International Society for the Advancement of Cytometry (ISAC) has highlighted the importance of including comprehensive methodological information to ensure data reproducibility and reliability. In line with this, Frontiers in Immunology now requires authors to submit a checklist for manuscripts that involve flow or mass cytometry. This checklist helps standardize the reporting process, improving the quality and transparency of published data. By doing so, we support scientific progress, making it easier for other researchers to replicate and validate experiments.

This form should be submitted with any manuscripts using flow or mass cytometry.

Sample/specimen/material description

☐ Total blood

☐ PBMCs

☐ Organ digests

Other RAW264.7 mouse macrophages （originate from cultures）

Did the samples suffer any treatment before or after incubation with the antibodies?

☐ Drug ____

☑ Cell permeabilization Fixation Buffer + Permeabilization Buffer

☐ Dye ____

☐ Propidium iodine

☐ Not applicable

Other Centrifugation washing, fixation, and permeabilization

Instrument and antibodies

Name of the Cytometer BD Accuri C6 Flow Cytometer (BD Company, USA)

**Antibodies and targets**	**Fluorochrome/ Metal**	**Catalog number/Company**
e.g. anti-CD4	FITC	Cat. XXX/ XXX ltd.
anti-mouse CD11b	FITC	E-AB-F1081C（Elabscience）

anti-mouse CD86	PE	E-AB-F1081C（Elabscience）
anti-mouse CD206	APC	17-2061-82（eBioscience）
Rat IgG2b, kappa Isotype control	FITC	400605（Biolegend）
Rat IgG2a, kappa Isotype control	PE	400501（Biolegend）
Rat IgG2b, kappa Isotype control	APC	400611（Biolegend）

Data analyses

1. Name of the software_____________________________________
2. Reference gating strategy in the manuscript or supplementary material

Gating strategy in (eg Figure X) ___________________________________
